# A study on the implementation of dual career at European higher education institutions: the student-athletes' and experts' views

**DOI:** 10.3389/fspor.2025.1507951

**Published:** 2025-02-25

**Authors:** Pascal Izzicupo, Sofia Serafini, Iris Prestanti, Andrea Fusco, Beatrice-Aurelia Abalasei, Tara Alonso del Hierro, Burak Çalışkan, Angela Di Baldassarre, Håkon Ege, Antonio J. Figueiredo, Barbara Ghinassi, Higinio González-García, Ionut Onose, Raluca-Mihaela Onose, Matteo Perissinotto, Amaia Ramírez-Muñoz, Antonio Sánchez-Pato, Nemanja Stanković, Nenad Stojiljković, Mojca Doupona, Laura Capranica

**Affiliations:** ^1^ Department of Medicine and Aging Sciences, University “G. D’Annunzio” of Chieti-Pescara, Chieti, Italy; ^2^ Faculty of Physical Education and Sports, “Alexandru Ioan Cuza” University of Iasi, Iasi, Romania; ^3^ Faculty of Health Science, Universidad Internacional de La Rioja (UNIR), La Rioja, Spain; ^4^ Collective Innovation AS, Oslo, Norway; ^5^ Research Unit for Sport and Physical Activity, Faculty of Sport Sciences and Physical Education, University of Coimbra, Coimbra, Portugal; ^6^ European Athlete as Student Network, Ghaxaq, Malta; ^7^ Department of Innovative Technologies in Medicine and Dentistry, University “G. D’Annunzio” of Chieti-Pescara, Chieti, Italy; ^8^ Department of Physical Education and Health, Faculty of Education, Universidad Internacional de La Rioja (UNIR), TECNODEF Research Group, Logroño, Spain; ^9^ OKKAM srl, Trento, Italy; ^10^ Faculty of Health, Universidad Internacional de La Rioja (UNIR), NÌKE Research Group, Logroño, Spain; ^11^ Universidad Internacional de La Rioja (UNIR), Vice-rectorate for Research, NÌKE Research Group, Logroño, Spain; ^12^ Faculty of Sport and Physical Education, University of Niš, Niš, Serbia; ^13^ Department of Sport Sociology and History, Faculty of Sports, University of Ljubljana, Ljubljana, Slovenia; ^14^ Department of Movement, Human and Health Sciences, University of Rome Foro Italico, Rome, Italy

**Keywords:** dual career, student-athletes, policies, programmes, sport and education, support entourage

## Abstract

**Introduction:**

The holistic development of elite athletes is *a priori*ty within European sports policies, necessitating a coordinated approach to dual career support. This study evaluated the perspectives of both university experts and student-athletes (S-As) on various dual career aspects, aiming to provide actionable insights for improving policies within European higher education institutions (HEIs).

**Materials and methods:**

Data was collected through an online survey tailored for HEI experts and S-As across multiple countries. A total of 46 HEI experts and 321 S-As responded to the survey. The role of the country of origin on each dual career aspect for S-As was investigated using a MANOVA, followed by an ANOVA and *post hoc* analyses using Tukey's test when an effect emerged. The data from HEI experts and comparisons between S-As and HEI experts were handled descriptively due to the violation of assumptions of homogeneity of variances and sufficient sample size.

**Results:**

The study revealed significant trends and disparities in the availability and quality of support services. In particular, logistic, and financial support, and other support/policies areas showed a significant effect for S-As countries of origin, with Romanian and Serbian S-As generally reporting better scores and Italian and Spanish worse. In general, HEI experts rated dual career provision areas more favorably than S-As.

**Conclusion:**

This study underscores the importance of integrating both HEI expert and S-As' perspectives to develop effective dual career policies. Tailored interventions and enhanced communication about available resources are crucial for improving the dual career experiences of S-As across Europe.

## 1 Introduction

The holistic development of elite athletes is *a priori*ty in sports policies of the European Parliament and globally (1–6), as it is essential not only for their immediate success but also for their long-term career prospects beyond athletics. The European Union (EU) is a geopolitical entity made up of 27 member states with different languages, cultures, regulations, and laws, retaining full authority over education and sports policies. Consequently, the EU implements a non-directive strategy aimed at fostering and facilitating coordinated intergovernmental collaboration to ensure a unified approach to development in these sectors (1–4, 7). In particular, the Bologna Process and the European Community Action Scheme for the Mobility of University Students (ERASMUS) have fostered structured cooperation among European higher education institutions, enhancing student, teacher, and staff mobility, standardizing degree accreditation and duration, unifying curricula content and teaching methods, and streamlining the credit transfer and accumulation system (ECTS) (8–10). In 2010, a seminal study on the implementation of policies for the combination of sports and academic careers (e.g., dual career, DC) across Europe (11) identified four main approaches: (1). State-centric regulation, with government legislation or statutory regulations imposing responsibilities on higher education institutions (HEIs) to provide flexible academic paths for student-athletes (S-As); (2). State as a sponsor/facilitator, with states promoting formal agreements to meet the S-As' educational needs; (3). National Sporting Federation/Institute as an intermediary, with national governing or sports bodies negotiating with HEIs flexible academic paths for S-As. (4). Laisser-faire/No Formal Structures, with sports bodies and HEIs as distinct and separate domains, thus requiring agreements individually negotiated, when possible, which can impair the holistic development of S-As. The publication of key documents, such as the EU Guidelines on Dual Careers of Athletes (12), along with more specific guidelines for universities and governing bodies (13, 14) has significantly advanced DC policies and provisions at the HEI level. Nonetheless, despite these efforts, the fragmented nature of dual career policies highlighted by Aquilina and Henry in 2010 remains largely unchanged. Many European countries still lack unified and comprehensive frameworks to fully support dual career development, resulting in significant variability in the availability and quality of DC services across the continent. Additionally, European studies examining minimum quality requirements for DC services (15) and qualifications related to DCs in sports (16) have further contributed to the DC progress. However, the European DC scenario remains fragmented and S-As still face considerable challenges in balancing their academic and sports careers due to limited or non-existent provision of assistance/tutorship, curricula requirements, financial support, logistic support, social support, and DC policies as aspects, which are deemed crucial for the holistic development of S-As (17). To ensure that the S-As academic and athletic needs are effectively met, both the opinion of HEIs employee/experts and S-As can offer important insights to monitor the implementation and maintenance of DC policies and services at HEIs (12–14, 18). Whilst the S-As views can highlight specific gaps and areas needing improvements to make the support systems relevant and effective, the opinions of HEI experts can provide a critical perspectives of institutional policies and support systems through their perceived relevance and the feasibility of DC implementation (17–19). Furthermore, the collaborative approach between HEI experts and S-As of different countries could identify common trends and national specificities, leading to various and widely accepted co-constructed solutions for policy-making (13, 14, 17, 20, 21). Recently, the European ERASMUS + Collaborative Partnership approved the “FIND-ME: University Dual Career Opportunities (FIND ME—101134043)” project, which is based on the coordinated efforts of 6 HEIs with a solid DC experience from Italy, Romania, Serbia, Slovenia, and Spain, the European Athlete as Student (EAS) DC network, and two innovation companies aiming to structure an evidence-based European platform specifically tailored for dual career at HEI level. The countries included in this study were not selected based on their classification within the typology of Aquilina and Henry (11) but were instead determined by their membership in the FIND-ME consortium. As such, their organization type within the typology is incidental. Therefore, the present study aims to: (i) Compare the opinions of HEI experts on DC provisions across different countries; (ii) Compare the perceptions of S-As on DC provisions across different countries; (iii) Identify similarities and differences between HEI experts' and S-As' perceptions of DC provisions, highlighting potential gaps in support services. These objectives were pursued through a survey evaluating multiple aspects of dual career support, in line with the recommendations of the EU Guidelines on Dual Careers of Athletes (12). Given the lack of prior research comparing the opinions of HEI experts and S-As on dual career provisions, the present study adopts an exploratory approach. The aim is to collect and analyze data to identify trends, similarities, and differences in their perceptions without formulating specific *a priori* hypotheses.

## 2 Materials and methods

All procedures were in accordance with the 1964 Helsinki Declaration for research involving human participants, including its later amendments or comparable ethical standards (Ethics Board of Universidad Internacional de La Rioja, Code: PI062/2024). Written informed consent was assumed with the completion of the online survey, which was chosen to provide time and geographic flexibility, as well as to facilitate multimedia integration and self-administration (22).

### 2.1 The instruments

Two questionnaires were developed based on validated instruments previously developed during ERASMUS + Sport projects specifically tailored for HEI experts and S-As, yet addressing common items on the actual availability, quality and potential implementation of DC policies and provisions at their HEI experts (14, 17, 18, 23). In particular, the FIND ME questionnaire for HEI experts was based on the More Than Gold instrument (14, 17, 18) and consisted of 31 questions organized into two sections. The first section (13 questions) gathered information on the country, the HEI and its academic areas, the number of degrees offered at the bachelor's, master's, and PhD levels, the respondent's academic position and DC role (e.g., tutor, academic referent, consultant, or other), the university website, and the availability of internal DC regulations in English. The second section (18 questions) assessed the current status of six thematic DC areas at the university, using a 5-point Likert scale to rate their quality from 1 (non-existent) to 5 (outstanding), and implementation status from 1 (not planned) to 5 (ongoing), as well as the expected time for implementation. These six thematic areas included logistic support, which refers to logistics and facilities, including access to sports and academic facilities, as well as housing options. Assistance and tutorship encompass all forms of assistance aimed at meeting S-As' needs, such as tutoring and psychological support. Curriculum requirements involve the modes of delivery for academic courses and examinations, including distance learning options, customized study plans, ECTS recognition, and other strategies. Social support includes initiatives promoting and recognizing dual career efforts and achievements of S-As, such as seminars, public campaigns, and recognition programs. Financial support covers financial assistance, such as scholarships, tuition fee waivers, and other forms of funding. Lastly, other forms of support and policies address additional aspects such as the establishment of dual career observatories, national legislation, and special contingents for athletes. Based on the ESTPORT instrument (23), the FIND ME questionnaire for S-As encompassed two sections for a total of 60 questions for the collection of the S-As' perceptions of DC programmes offered at their HEIs. The first section included demographic information, such as nationality, age, gender, practiced sport, competitive level, the academic level and major, and the type of their HEI (e.g., private, public) and academic programme (e.g., on-site or online). The second section collected opinions about the six thematic areas for DC included in the instrument administered to the HEI experts (e.g., logistic support, assistance/tutorship, curricula requirements, social support, financial support, and other supports/DC policies). This section used a 5-point Likert scale from 1 (lowest value) to 5 (highest value).

### 2.2 Recruitment

Through their national and European non-FIND ME networks, the FIND-ME team identified and invited potential respondents, informing them that participation was voluntary and anonymous, and that they could withdraw from the study at any time without providing a reason. A link to the online survey was provided, and informed consent was assumed upon completion of the survey. The survey opened on March 25, 2024, and closed on June 15, 2024. To increase the response rate for online surveys encompassing more than 20 items, reminders were sent every two weeks (22). Given the significant heterogeneity in dual career provisions across Europe—both between countries, as highlighted by Aquilina's typology, and within countries, where universities may adopt diverse strategies or implement national guidelines differently, even when national legislation exists—the sampling strategy adopted was flexible. Potential respondents were identified through three main approaches: (i) Direct contact with individuals responsible for dual career initiatives, based on the existing knowledge and professional networks of project partners; (ii) Leveraging the EAS network, which maintains an up-to-date database of European professionals working in dual career support; (iii) Requesting universities to indicate the appropriate office or individual responsible for dual career provisions. Eligible participants were defined as any individual serving as an internal reference point for dual career matters within their university, including faculty members, researchers, or administrative staff involved in dual career support or policy implementation. Exclusion criteria were applied to exclude individuals without a formal or recognized role in dual career initiatives within their institutions, ensuring that all respondents had direct experience or responsibilities related to dual career provisions.

### 2.3 Characteristics of the sample

Characteristics of the sample are showed in Table 1. A total of 69 responses were collected for the HEI experts' questionnaire, with 24 responses subsequently excluded due to duplicate (*n* = 1) answers erroneously provided by S-As (*n* = 2) or by university representatives indicating that no DC policy was in place (Bosnia-Herzegovina: *n* = 2; Kosovo: *n* = 2; Italy: *n* = 5; Latvia: *n* = 2; Romania: *n* = 8; Serbia: *n* = 2). Most of the remaining 45 responses were provided by HEI experts holding DC responsibilities (65.2%), followed by DC tutors (15.2%), DC consultants (15.2%), and other roles such as Olympic Committee Assistant, Sports Delegate, and member of the institutional DC commission (2.1%). The countries with the highest HEI representation were Italy (50.0%), Romania (15.2%), and Spain (13.0%), followed by Serbia (8.7%) and Slovenia (4.3%), with additional contributions from Croatia, Latvia, Poland, and Portugal (2.1%). Only 11 HEIs provide DC regulations in English (Italy: *n* = 4; Romania: *n* = 5; Serbia: *n* = 1; Spain: *n* = 1).

**Table 1:** Distribution of HEI experts and S-As by country.

Country	Number of HEI experts and participating universities	Number of S-As
Italy	22	122
Romania	7	52
Serbia	4	44
Slovenia	2	17
Spain	6	58
Non-Find Me Countries	4	28
Total	45	321

From the 329 responses initially gathered from S-As, 8 were excluded due to erroneous answers provided by non-athletes (*n* = 6) or former athletes (*n* = 2). From the remaining 321 responses, most were Italian S-As (*n* = 122, 38%), followed by Spanish (*n* = 58, 18.1%), Romanian (*n* = 52, 16.2%), Serbian (*n* = 44, 13.7%), Latvian (*n* = 18, 5.6%), Slovenian (*n* = 17, 5.3%), Kosovarian (*n* = 9, 2.8%), and English (*n* = 1, 0.3%). There was a balanced proportion for sport typology (individual sports: *n* = 161, 50%; team sports: *n* = 160, 50%) and gender (males: *n* = 189, 58.9%; females: *n* = 132, 41.1%). Most of the participants were semi-professional athletes (*n* = 149, 46.4%), followed by their professional (*n* = 113, 35.2%) and amateur (*n* = 59, 18.4%) counterparts.

### 2.4 Data analysis

The sample sizes for S-As and HEI experts were not homogeneous due to the varying numbers of universities present in each country, ranging from three public universities in Slovenia and more than 90 in Italy, Romania, and Spain. Considering that a single approach to the analysis would have violated the assumptions of homogeneity of variances and sufficient sample size, linear statistics were applied to S-As' data whereas a descriptive approach was applied to HEI experts' data. To investigate the role of the country of origin of the S-As, a MANOVA was applied to each of the six thematic areas included in the survey, with the respondents' country of origin (e.g., Italy, Romania, Serbia, Slovenia, Spain, and Non-Find ME Countries) as the independent variable. To control for Type I error, Bonferroni corrections were applied to the significance levels of the six separate MANOVAs, while Pillai's Trace was used for the MANOVAs between countries, as it guarantees high robustness in presence of violation of the assumptions of homogeneity of covariance matrices and normality. For significant differences (*p* < 0.05), univariate ANOVAs were conducted on individual items within each area, with Bonferroni corrections applied to control for Type I errors. *post hoc* analyses using Tukey's test were conducted to further explore significant differences between groups. All statistical analyses were computed using JASP Team (2023). JASP (Version 0.18.1).

## 3 Results

### 3.1 University experts' opinions of the DC provision

For each considered country, Supplementary Table 1 reports the items of the six dual career areas at HEIs including the percentages of actual presence and the means and standard deviations of the HEI experts’ quality ratings, and the percentages of planned implementation and the expected years, respectively. In general, HEI experts reported the presence of DC services ranging from 88 ± 5% (Serbia) and 63 ± 15% (Spain). The implementation plans are expected to be completed from 2024 and beyond 2027, with the Serbian HEIs reporting the highest percentages (range: 50%–100%, 91 ± 14%), and one of the Slovenian Universities (50%) planning the implementation of only the education facilities. Overall, HEIs from the other countries reported limited implementation plans (Italy: 49 ± 16%; Romania: 58 ± 12%; Spain: 21 ± 14%; and Non-FIND Me countries: 78 ± 24%).

Logistic support showed the highest presence and quality ratings, with higher values were reported for educational facilities (range: 83%–100%; 3.3–5.0 pt), sport facilities (range: 75%–100%; 3.0–5.0 pt), and economic investments in facilities (range: 83%–100%; 2.5–4.4 pt) compared to accommodation for S-As (range: 37%–100%; 2.0–4.3 pt), which showed the lowest presence for Italy (37%), and the lowest quality ratings for Spain (2.0 pt). The implementation plans for the items included in logistic support showed a high variability (Italy: 69 ± 13%; Romania: 71%; Serbia: 94 ± 13%; Slovenia: 13 ± 25%; Spain: 17 ± 13%; Non-FIND ME countries; 69 ± 13%).

Regarding the assistance and tutorship area, the tutorship/mentorship item was more prevalent in Spain, Non-FIND ME countries and Italy (range: 96%–100%, 4.3–4.6 pt) compared to Romania, Serbia, and Slovenia (range: 50%–75%, 3.5–5.0 pt). But for Serbia and Romania (range: 50%–71%, 2.0–2.8 pt), individual programme showed a high presence and quality (range: 96%–100%, 3.5–4.4 pt). Slovenia and Non-FIND ME countries reported a full integration of dual career in academic department, sports or professional services (quality range: 3.3–3.5 pt) and psychological support (quality range: 2.5–3.5 pt), whereas the relative picture for the other countries ranged from 83% (Spain) to 57% (Serbia) with 2.3–3.7 pt quality ratings for integration of dual career in academic department, sports or professional services, and ranged from 77% (Italy) to 67% (Spain) with 3.9–2.7 pt quality ratings for the psychological support. Except for Non-FIND ME countries (100%, 3.3 ± 0.5 pt), dual career proactive programmes showed a limited presence (range: 30%–77%) with a quality range of 3.3–3.5 pt. The implementation plans for the items included in the assistance/tutorship area also showed high variability (Italy: 52 ± 3%; Romania: 54 ± 16%; Serbia: 100%; Slovenia: 0; Spain: 24%; Non-FIND ME countries; 75 ± 29%).

For the curricula requirements area, individualized study plans were not reported for Serbia, with the other countries showing a presence ranging from 86% (Romania) to 50% (Slovenia), with a quality rating ranging from 3.8 pt (Spain) to 5.0 pt (Slovenia). While Slovenia and Non-FIND ME countries indicated a full availability of distance learning (quality range: 3.5–4.3 pt), the other countries showed a lower presence (range: 59%–86%) with a quality ranging from 2.7 pt (Romania) to 4.0 pt (Serbia). Low presence of recognition of ECTS for the sport career was reported (0%–75%) with a quality ranging from 2.7 pt (Romania) to 5.0 pt (Spain). Similarly, except for Serbia (100%, 3.0 pt), untraditional learning strategies showed a limited presence (range: 14%–75%) with a quality range of 2.0–4.0 pt. The implementation plans of the items included in the curricula requirements area resulted 42 ± 10%, 64 ± 8%, 92 ± 13%, 0%, 13 ± 9%, and 69 ± 13% for Italy, Romania, Serbia, Slovenia, Spain, and Non-FIND ME countries, respectively.

For the social support area, in general HEIs provided publicity for their S-As representing the university (range: 83%–100%, 3.0–4.7 pt). While HEIs in Serbia and Non-FIND ME countries declared to organize local/international seminars, workshop, meeting on dual career issues (100%, 2.7–4.0 pt), the other countries showed lower presence (range: 50%–71%, 3.5–4.0 pt). Except for Serbia (100%, 2.7 ± 0.6 pt), the other countries showed lower presence of an institutional dual career committee (range: 50%–63%, 3.0–4.1 pt). Only the Non-FIND ME countries reported providing publicity of S-As' characteristics for labor market, whereas the relative picture for the other countries range 43%–75% with a mean quality ranging from 2.3 pt. (Serbia) to 3.7 pt (Romania). Peer to peer was fully implemented at HEIs in Romania, Serbia, and Non-FIND ME countries (quality range: 2.7–3.7 pt), whereas lower presence emerged for the other countries (range: 50%–73%, 3.5–5.0 pt). Except for Non-FIND ME countries (100%, 3.3 ± 1.0 pt), the others tended not to organize seminars, workshop, meetings with parents and coaches (range: 40%–75%, 2.5–3.7 pt). The HEIs from Serbia and Non-FIND ME countries reported the highest frequency of implementation plans (92 ± 13%) of the items included in the social support area, whereas the relative picture for Italy, Romania, Slovenia, Spain was 57 ± 12%, 64 ± 8%, 0%; 33 ± 10%, respectively.

Overall, lower values emerged for the items included in the financial support area (range: 9%–64%, 2.7–4.0 pt), especially evident for the lack of scholarships for S-As in Serbia and Slovenia, the remission of tuition fees for S-As in Slovenia, and salary in Romania, Slovenia, and Spain. Except for Serbian HEIs (81 ± 24%), the implementation plans of the items included in the financial support area presented low frequency of occurrence (Italy: 29 ± 12%; Romania: 42%; Slovenia: 0; Spain: 17 ± 13%; Non-FIND ME countries; 69 ± 47%).

Only the HEIs from Serbia and Non-FIND ME countries declared a full provision of the items included in the other supports/DC policies area (quality range: 2.7–4.0 pt), whereas no observatory of the application of the dual career statute was reported for Slovenia. In general, higher presence emerged for national legislation and lower for special access contingent for actual or former elite athletes. Also, for the implementation plans of the items included in the other supports/DC policies area the HEIs from Serbian (100%) and Non-FIND ME countries (92 ± 14%) presented the highest frequency of occurrence, whereas lower values emerged for Italy: 41 ± 10%; Romania: 43%; Slovenia: 0; Spain: 17 ± 17%.

### 3.2 S-As opinions

For all the considered six thematic areas of DC Box's M test indicated a violation of the assumption of homogeneity of covariance matrices. Additionally, the Shapiro–Wilk test indicated a violation of multivariate normality. Supplementary Table 2 reports the items of the six dual career areas at HEIs including their percentages of the presence and the means and standard deviations of the S-As' quality ratings.

#### 3.2.1 Logistic support

The country of origin showed a significant effect on the opinions regarding logistic support [Pillai's Trace = 0.24, Approx. *F*_(20, 1260.00_) = 4.11, *p* < 0.001]. But for the quality of economic investment for university facilities, the other dimensions of the students' ratings of perceived quality of logistic support services showed significant effects as reported in Supplementary Table 2. For access to educational facilities, a significant effect was found, with *post hoc* analyses revealing that Italian students perceived lower quality compared to Romanian students. Regarding the quality of sport facilities, a significant effect was found with *post hoc* analyses underlining that Romanian reported higher quality ratings with respect to their Italian and Spanish counterparts. Finally, for the quality of accommodation facilities for S-As, the ANOVA results indicated a significant effect. *post hoc* analyses revealed that Romanian students rated the quality higher compared to both Italian and Spanish students. Additionally, Romanian students rated the quality higher compared to Slovenian and Non-Find ME students. Furthermore, Serbian students reported higher quality ratings compared to Spanish students.

#### 3.2.2 Assistance/tutorship

A country-related effect of the opinions regarding assistance/tutorship emerged [Pillai's Trace = 0.22, Approx. *F*_(25, 1575.00)_ = 2.85, *p* < 0.001]. However, further comparisons between countries showed no differences for the various items in this category.

#### 3.2.3 Curricula requirements

A country-related effect was found for the S-As' opinions regarding curricula requirements [Pillai's Trace = 0.11, Approx. *F*_(20, 1260.00)_ = 1.70, *p* = 0.03]. However, further analysis showed no effects of the perceived quality of individual study plans, distance learning, and recognition of ECTS for the sport career, whereas the initial effect emerging for the quality of available untraditional learning strategies (e.g., creating digital portfolios, using social networks) was not maintained after applying the Bonferroni correction.

#### 3.2.4 Social support

The country of origin showed a significant effect on the opinions regarding social support [Pillai's Trace = 0.26, Approx. *F*_(30, 1570.00)_ = 2.93, *p* < 0.001]. Despite the initial effects observed for local to international seminars, workshops, and meetings on up-to-date DC issues, and for publicity on S-As and their characteristics suitable for the labor market, the Bonferroni correction did not maintain any significant difference for these DC aspects. For countries, a significant effect was found for the quality of social support through an institutional DC committee, with *post hoc* analysis showing that Italian students reported lower ratings compared to Serbian students, but higher ratings compared to Spanish students. Furthermore, Romanian students reported significantly lower ratings compared to Serbian students, and Serbian students reported significantly higher rates compared to Spanish students. For peer-to-peer support, a significant effect was found with *post hoc* analyses revealing lower ratings for Italian students compared to Serbian students, who reported higher ratings compared to Spanish students and Non-Find ME students. Additionally, an effect was found for seminars, workshops, and meetings with parents and coaches, with Italian students reporting lower ratings compared to Serbian students, who reported higher values compared to Spanish students (Supplementary Table 2).

#### 3.2.5 Financial support

The country of origin proved had an effect on the opinions regarding financial support [Pillai's Trace = 0.30, Approx. *F*_(20, 1260.00)_ = 5.05, *p* < 0.001]. An effect was found for the quality of scholarships, with Italian students reporting lower ratings compared to their Romanian and Serbian counterparts. For the remission of tuition fees for S-As, an effect was found, with Serbian students reporting higher ratings compared to Romanian and Spanish students. Regarding the salary for S-As, an effect emerged, with Serbian students reporting higher ratings compared to Italian, Romanian, Slovenian, Spanish, and Non-Find ME students. Furthermore, differences were maintained also for Romanian students reporting higher ratings compared to Spanish students (Supplementary Table 2).

#### 3.2.6 Other support/Dc policies

The country of origin had an effect on the opinions regarding other support/DC policies [Pillai's Trace = 0.16, Approx. *F*_(15, 945.00)_ = 3.45, *p* < 0.001]. The perceived quality of a sport observatory (controlling and monitoring the application of the DC statute) showed an effect, with Serbian students showing higher ratings compared to their Italian, Romanian, and Spanish counterparts. For the quality of national legislation, an effect was found, with Serbian students reporting higher ratings compared to Italian, Romanian, Spanish, and Non-Find ME students. For the quality of special access contingents (reserved for actual or ex-high sport performance practitioners), an effect was found, with Serbian students showing higher ratings compared to Italian, Romanian, Spanish, and Non-Find ME students (Supplementary Table 2).

### 3.3 Comparison between HEI experts and S-As

**Figure 1:**
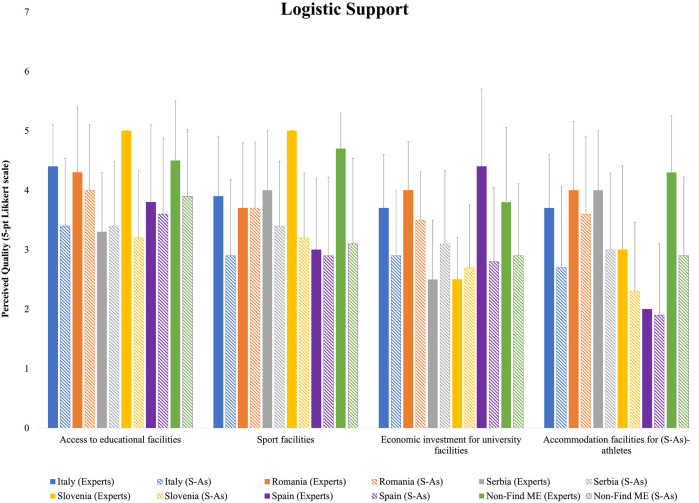
Means and standard deviations of the national HEI experts’ and S-As’ perceived quality for logistic support ratings.

On average, S-As rated the quality of logistic support lower than their academic counterparts, with higher ratings for access to educational facilities and lower ratings for accommodation facilities (Figure 1). Closer agreement between HEI experts and S-As was observed regarding access to educational facilities for Romanian and Spanish respondents, while Serbian S-As provided slightly higher evaluations than the HEI experts. Romanian and Spanish respondents also offered similar evaluations for sports facilities, whereas Serbian S-As rated them even higher than their expert counterparts. For accommodation facilities, Romanian and Spanish respondents once again showed closer alignment with HEI expert evaluations.

**Figure 2:**
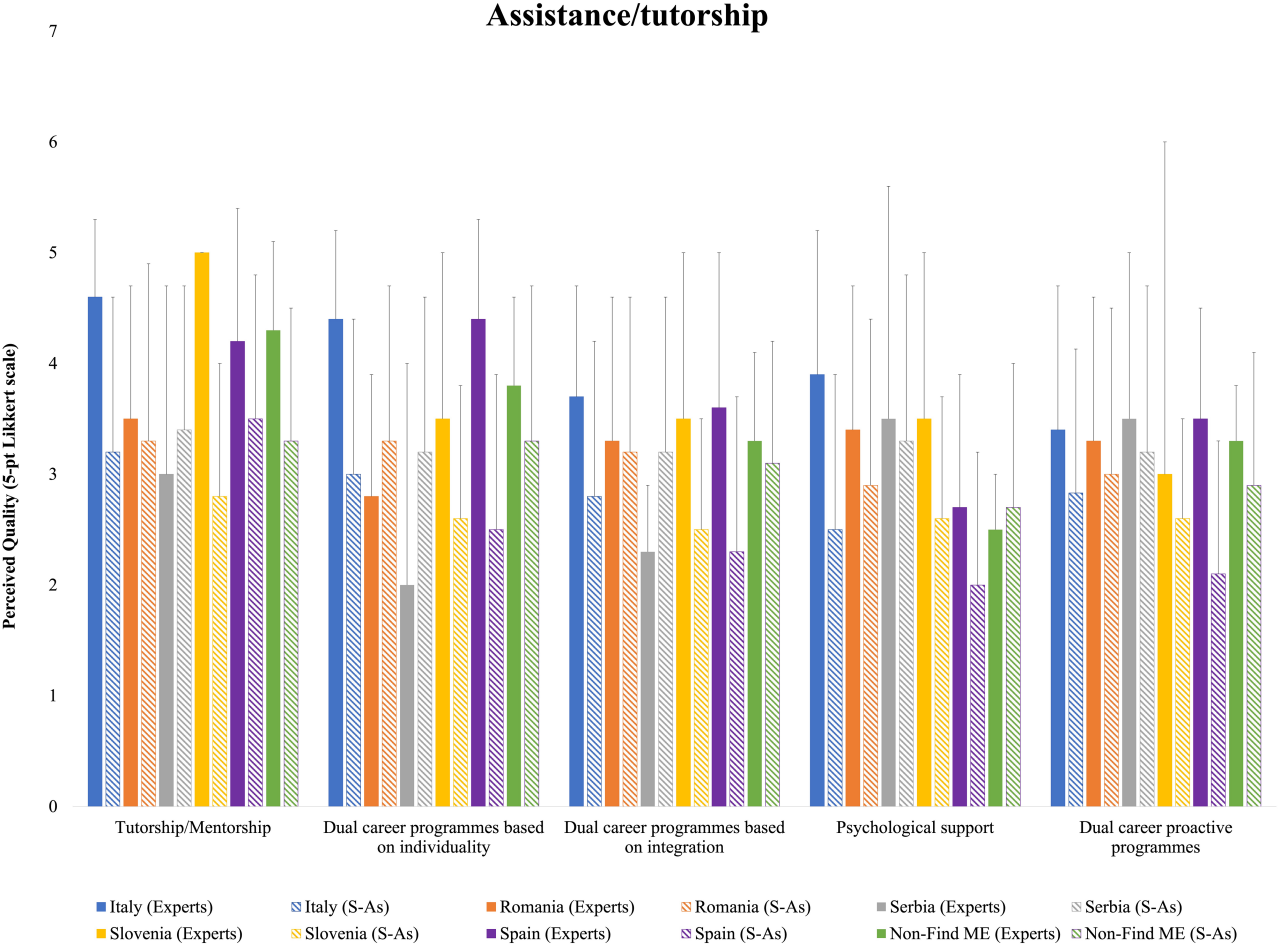
Means and standard deviations of the national HEI experts’ and S-As’ perceived quality for assistance/tutorship support ratings.

Figure 2 shows the average quality of HEI experts and S-As for assistance and tutorship. Except for Romanian HEI experts and S-As showing similar ratings and Serbian S-As showing higher ratings than HEI experts for the items of the assistance/tutorship area, experts of the other countries generally showed higher quality ratings with respect to their S-A counterparts. For DC programmes based on individuality, Romanian and Serbian HEI experts reported lower quality ratings with respect to those of S-As, whereas the opposite picture emerged for Italian, Slovenian, Spanish and Non-FIND ME HEI experts. Mixed results were found for DC programmes based on integration, with Serbian S-As reporting higher evaluations than the HEI experts. Comparable quality ratings for psychological support emerged for HEI experts and S-As in Serbia and Non-Find ME countries, but higher especially for Italian, Slovenian, and Spanish HEI experts. For DC proactive programmes, lower quality ratings for S-As with respect of those of the respective HEI experts’ counterparts.

**Figure 3:**
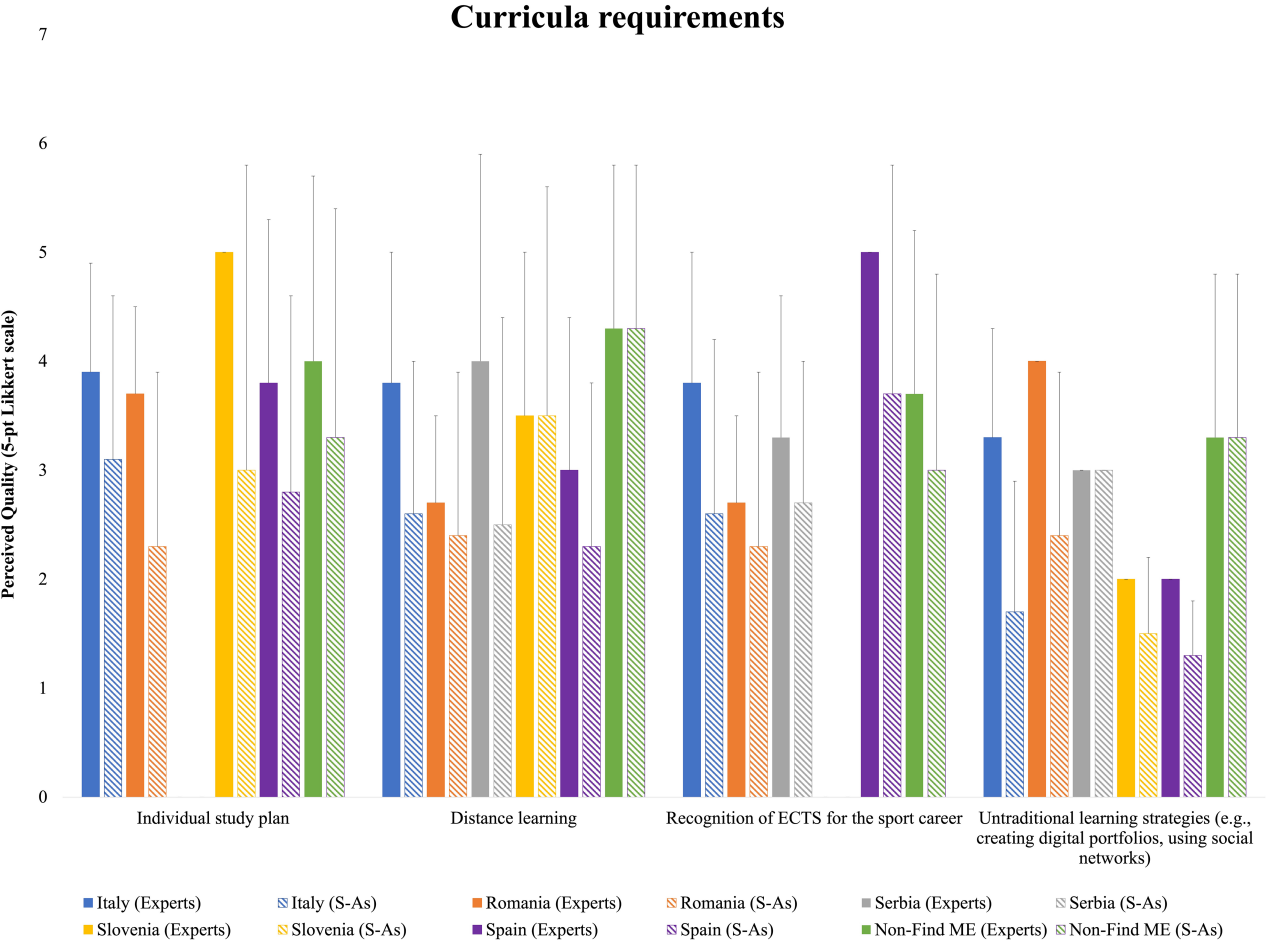
Means and standard deviations of the national HEI experts’ and S-As’ perceived quality for curricula requirements ratings.

Except for a few cases, HEI experts generally rated the quality of curricular requirements higher than their respective S-A counterparts (Figure 3). The evaluation of the Individual Study Plan and the recognition of ECTS for sports careers were consistently rated higher by experts. Regarding distance learning, closer evaluations were observed among Romanian, Slovenian, and Non-Find-ME respondents. For untraditional learning strategies, Italian and Romanian HEI experts rated considerably higher than S-As, while Serbian and Non-Find-ME respondents provided similar evaluations.

**Figure 4:**
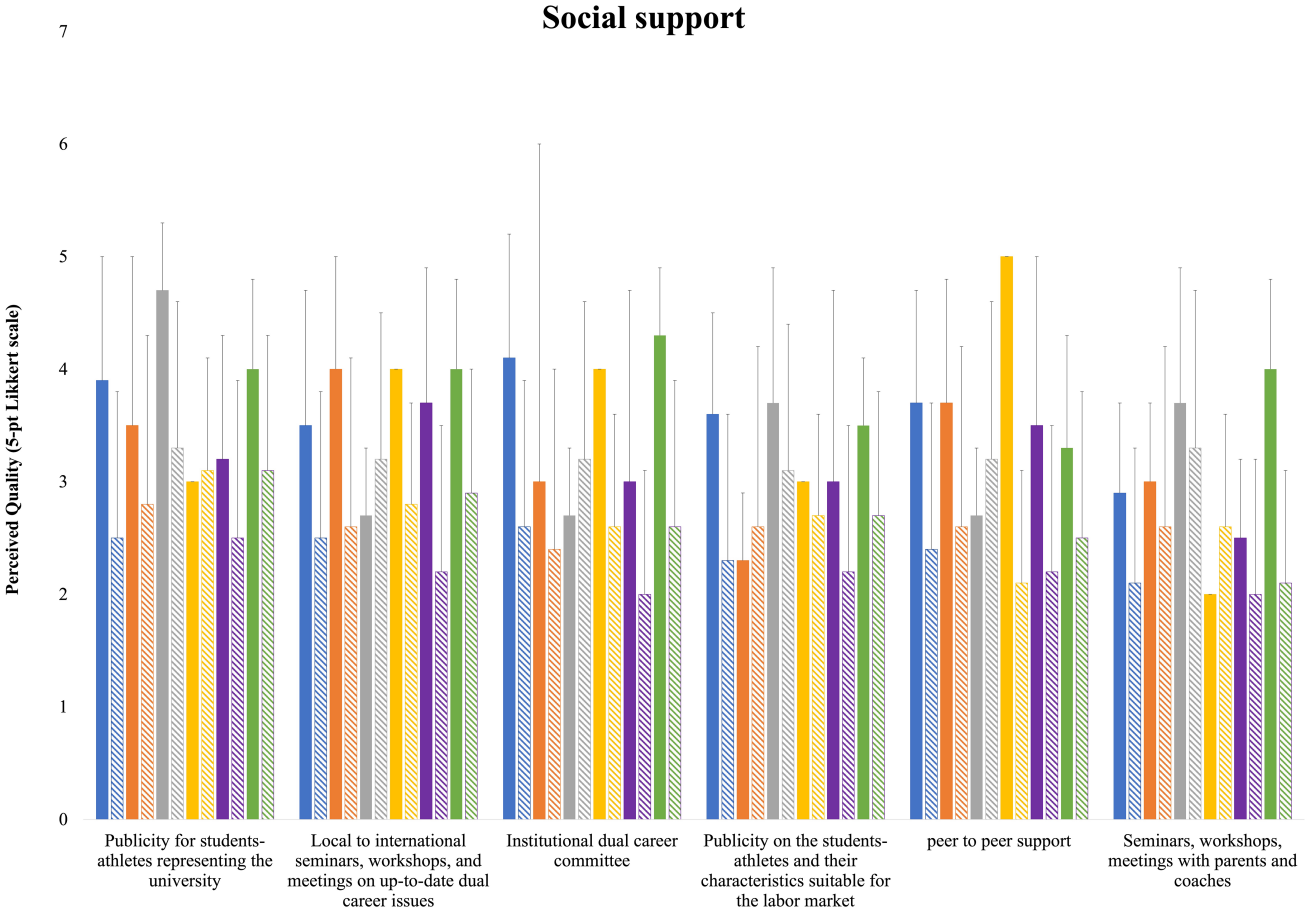
Means and standard deviations of the national HEI experts’ and S-As’ perceived quality for social support ratings.

Figure 4 shows the Except for average quality of HEI experts and S-As for social support. Slovenian respondents, HEI experts generally tended to rate the publicity for S-As representing the university higher than their S-A counterparts. A larger discrepancy was evident for Italian and Serbian respondents. Regarding local to international seminars, workshops, and meetings on up-to-date DC issues, only Serbian S-As provided higher ratings than their expert counterparts, while Romanian and Spanish respondents exhibited the largest discrepancy between S-As and HEI experts. Serbian S-As also rated the institutional DC committee higher than HEI experts, whereas Non-Find-ME HEI experts rated it considerably higher than their S-As. For publicity about S-As and their characteristics suited for the labor market, only Romanian S-As rated the service better than HEI experts, although Slovenian respondents provided closely aligned evaluations. Serbian S-As once again rated peer-to-peer support better than HEI experts, with the largest discrepancy between S-As and HEI experts observed for Slovenian respondents. Regarding seminars, workshops, and meetings with parents and coaches, Slovenian S-As rated them higher than HEI experts, while Romanian, Serbian, and Spanish respondents showed closely aligned evaluations (Figure 4).

**Figure 5:**
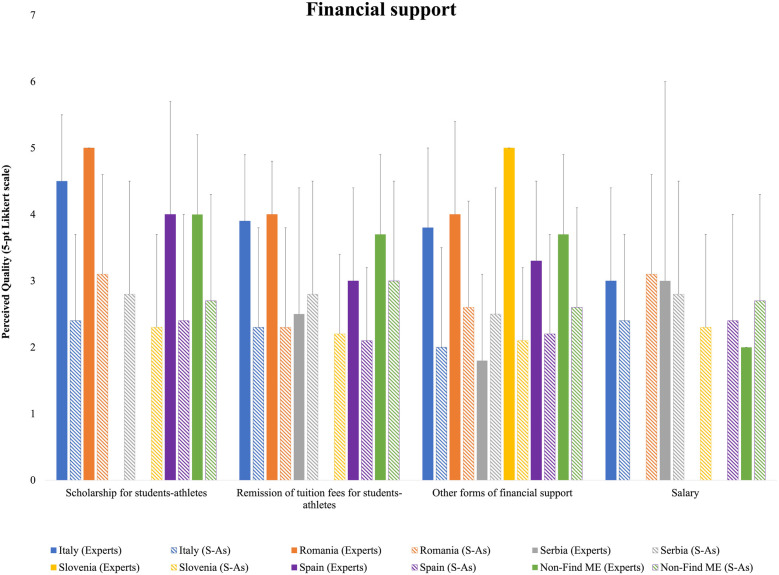
Means and standard deviations of the national HEI experts’ and S-As’ perceived quality for financial support ratings.

Regarding the category Financial support, Serbian and Slovenian HEI experts did not provide evaluations for scholarships, while the remaining respondents rated them higher than S-As, particularly the Italians. Regarding the remission of tuition fees, only Serbian S-As provided higher evaluations than HEI experts, whereas Italian and Romanian experts showed the largest discrepancies compared to their S-As counterparts. Similarly, Slovenian experts did not provide ratings in this category. Other forms of financial support were once again rated higher by Serbian S-As, while Italian and Slovenian HEI experts displayed the largest differences in ratings compared to their S-As. Finally, regarding salary evaluations, only Italian, Serbian, and Non-Find-ME HEI experts provided assessments, showing close alignment with Serbian respondents, but with higher ratings from Non-Find-ME S-As compared to HEI experts (Figure 5).

**Figure 6:**
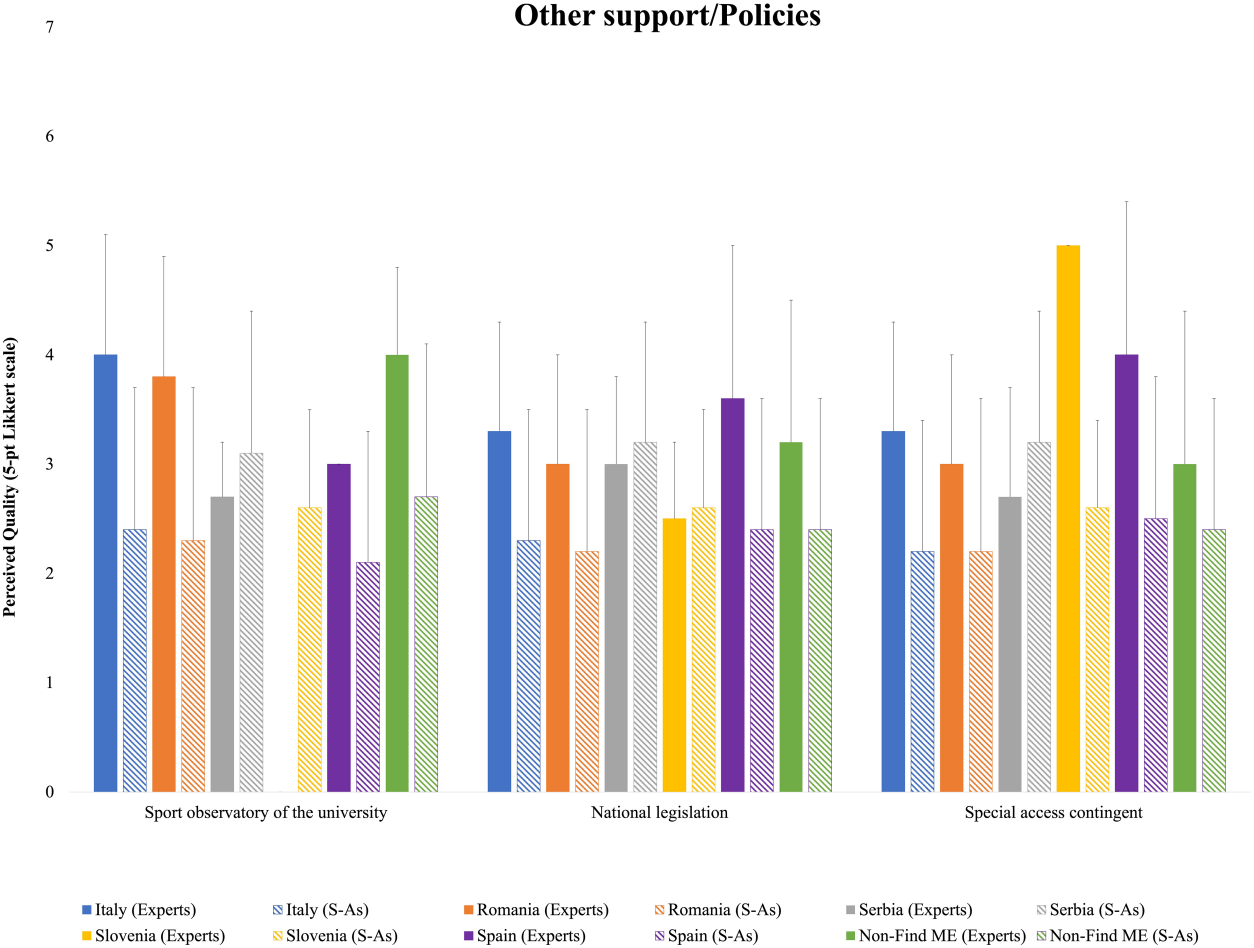
Means and standard deviations of the national HEI experts’ and S-As’ perceived quality for other support/policies ratings.

The remaining categories related to other forms of support and policies are shown in Figure 6. Apart from Serbian respondents, HEI experts generally rated these categories higher than S-As. The university's Sports Observatory rating showed a similar discrepancy between experts and S-As across all groups, except for Serbian respondents. Moreover, while Slovenian S-As provided a rating for this aspect, HEI experts marked it as unavailable. For National Legislation, higher ratings were attributed by HEI experts in all cases except Serbia, where S-As provided better evaluations, and Slovenia, where the values were nearly identical. Finally, for Special Access Contingency, the largest discrepancy between HEI experts and S-As was observed among Slovenian respondents, followed by Spanish and Italian respondents, whereas the differences were less pronounced for Romanian and Non-Find-ME countries.

## 4 Discussion

In reporting the perspectives of both HEI experts and S-As on DC aspects, the present explorative study aimed to: (i) compare the opinions of HEI experts on DC provisions across different countries; (ii) compare the perceptions of S-As on DC provisions across different countries; (iii) identify similarities and differences between HEI experts' and S-As’ perceptions of DC provisions, highlighting potential gaps in support services. These objectives were pursued to provide actionable insights for improving DC policies and provisions at European HEIs. In line with the literature (18), the main findings confirmed the differences in the provisions of DC services between and within the investigated countries. In addition, the surveys revealed not only the quality of the actual DC services, but also the ongoing or planned implementation expected in the next few years, which is a consequence of the firm European policies on raising the awareness and supporting the development of a DC discourse (4). Whilst S-As might base their evaluations on personal experience of practical usability and personal convenience of the received DC support, HEI experts have a broad view of institutional policies and support systems, focusing on the overall provision rather than specific daily usability (24). Furthermore, S-As may be less satisfied if they are aware of better facilities available elsewhere compared with the current support (25). On the other hand, HEI experts may evaluate based on realistic achievements within budgetary and policy constraints. Finally, both HEI experts and S-As may not be fully aware of all available resources for supporting and developing DC competencies, being DC support providers a not established and regulated profession within the European context (26, 27). Depending on the HEIs' and S-As' perspective taken, the results could be interpreted under the perspective of “a glass half empty or half full”. However, the identified nuances could help best support DC initiatives and assist HEIs and governments in implementing policies conducive to improving the holistic development of S-As as empowered citizens.

Logistic support resulted as the most prevalent service provided to S-As. Whilst a high-quality provision of educational facilities was expected, the opinions of both HEI experts and S-As highlighted the necessity for tailored interventions to enhance the DC experiences of S-As across different countries. The additional implementations declared to be ongoing or expected already in the next year are in line with the European quest for innovative educational tools, methods, and resources tackling inclusive and connected social challenges (28). Within this area, the provision of accommodation for S-As resulted the least available service, which is not surprising, as only an average of 15% of students in Europe live in student accommodations, despite the substantial gap between the demand and supply, and the high international student mobility supported by the ERASMUS programme (29, 30). European countries do not have an official mandate on the provision of affordable on-campus housing, which is primarily a concern of national and local policies and culture, yet it remains a crucial aspect of the academic experience of students. The present findings highlight differences of the S-As' perceptions on the available logistic support, with Romanian S-As consistently rating higher quality compared to their Italian and Spanish counterparts. In fact, HEIs in central and northern Europe provide more well-structured on-campus housing programmes with respect to their Mediterranean counterparts (29). While pinpointing the exact origins of the important differences between countries is beyond the aim of this study, it is plausible that socioeconomic factors, such as national income levels, public funding for higher education, and the effectiveness of policy implementation within countries play a crucial role. However, the significant cross-country differences suggest the need for a tailored approach to develop and implement integrated institutional strategies to improve the overall satisfaction and well-being of their students (29). In having strong, direct, and personal relationships with the athlete, tutors play a crucial supporting role of the S-As entourage (6, 31, 32). Following the publication of the EU Guidelines on DCs of Athletes, HEIs were urged to adopt new pedagogical models and to provide a tutoring system for their S-As (31). Despite the differences between and within countries, in general HEIs offer tutorship/mentorship and individualized programmes, and planned their implementation in the next years to meet the desires and needs of S-As. To note, no difference between countries emerged for S-As' perceived quality of tutorship, even though ample margin of amelioration is envisaged. The development process of S-As and the environment that supports them is complex and dynamic, with both contributing to each other's evolution (33). Due to a lack of a defined professional role and defined skills, at present tutors learn how to support students by addressing the various scenarios the S-As encounter to navigate effectively their sports and academic environments. To facilitate this process, there is a need to integrate and consolidate the cooperation between the academic, professional, and sports services and activities currently scattered across different agencies, units, departments, and institutions. This cooperation could help structuring individualized programmes ensuring suitable DC paths for each athlete, and to adopt a proactive attitude to anticipate future needs of the S-As (31). To foster both the academic and athletic development, a key factor is the creation of a cohesive and integrated support network that aligns with the individual needs of S-As. Interesting to note, S-As rated higher the quality of DC proactive programmes with respect to their HEI expert counterparts. In considering the development process of DC in the last decade, it is possible to speculate that university S-As might have experienced a limited support during the previous years, which made them particularly appreciate the sensible DC management and climate factors offered at their HEIs.

Notwithstanding the Bologna process, uniformity in curricula requirements still presents a challenge in the harmonization of European higher education systems due to the different regulations in place in the Member States (9) European Commission, 2012; European |Parliament, 2004, 2017). In this area, the HEI experts confirmed the differences between countries, with individual study plans, distance learning, and ECTS recognition for the sport career being more represented with respect to untraditional learning strategies. However, most of the HEI experts reported plans for implementation expected in the next years, in line with the European quest of increasing innovation and competitiveness, and the promotion of harmonization for an automatic and mutual recognition of the ECTS credits also earned outside the higher education, the Diploma Supplement, micro-credentials, and the spread of digital learning approaches associated with the COVID-19 pandemic (10). Harmonisation and interoperability depend on interlinkages between actors, policies and provisions, which lead to further interlinkages and interdependencies. In this respect, DC could be considered an innovative approach, with S-As valuing their DC skills for maintaining effective time management, self-motivation, and discipline during unexpected COVID-19 circumstances with respect to their non-athlete counterparts (34). Unlike other DC areas that are strongly influenced by individual university policies and national directives or laws, curricula requirements and assistance/tutorship relate closely to university activities that directly involve students and professors. Thus, the rather low S-As quality ratings of this area, with no differences between countries, indicate areas where alignment and improvements may be needed to enhance the DC experiences. Often, S-As are not fully aware of their role and the services available to them, which can be due to insufficient communication from the institutions or a lack of proactive engagement from the students themselves (20). This lack of awareness can lead to underutilization of available resources, resulting in a perceived uniformity in evaluations. It is essential to improve the visibility and accessibility of these support services to ensure that S-As can fully benefit from them (17, 35). According to the data collected from HEI experts and S-As, the social support area presents important margin for implementation through the organization of seminars, workshops and publicity of the S-As, in line with the Athlete365 Career + programme for Olympic athletes (www.olympic.org/athlete365/career). In establishing positive relationships with faculty and peers S-As build confidence, academic focus, and strong university connections. In this study, publicity for student-athletes representing the university provided higher presence and quality results for HEI experts with respect to their S-As counterparts. Whilst the American collegiate athletics association (NCAA) has traditionally been coupled with the academic mission of an institution that influence the social and cultural discourse of campus life, in the labour market companies engage elite sportspersons as testimonials for the promotion of their products and for creating meaning and value transfer (36, 37). In considering that a significant implementation is expected in the next years, the present findings indicate a need to co-create value through DC (18, 36). Much of the social support might be delivered through new channels (e.g., the publicity for S-As in the job market and their achievements), which can leverage the same social channels across various countries, contributing to the lack of differences in S-As opinions. Although parents and coaches represent crucial actors of the social support entourage of S-As (20, 38), the present findings highlight that HEIs pay a limited attention to engage them in seminars and workshops. Recently, a free online multilingual educational programme has been developed to empower parents of S-As athletes (https://edu.empatiasport.eu/it/), which could be used to organize seminars, workshop, meetings to bridge the gap between HEIs and the parents and coaches for establishing a cohesive supporting entourage of their S-As (17, 38). Financial investments and HEI funding systems differ tremendously across Europe, being closely associated with university policies, which are in turn constrained by the national regulatory framework (39). Although in Europe the average tuition amount of public HEIs is rather low when compared to that of HEIs from Anglo-Saxon, North and Latin American, and Northeast Asian countries, Continental and European Southern European countries spend less on financial student aid, often based on need-based (e.g., family or individual income) and merit-based (e.g., ECTS and grade point average) grants, or a combination of the two, with sport achievement receiving little or no consideration (39). Coherently, in the present study the area of financial support tended to present the lowest support, especially related to the salary for S-As. Furthermore, the national socioeconomic context and cultural background can influence the S-As' expectations. In countries with stronger economic support structures, S-As may expect more comprehensive financial aid, whereas in others, lower expectations might result in higher satisfaction with more limited resources. In being not financially independent when competing in nonrevenue-generating sports, S-As reported financial uncertainty and envisaged possible improvements, which would merit future attention from policy makers and future studies on the financial support S-As receive from government, HEIs, families, and sports and private sectors (20).

According to (11), the countries included in our study have different approaches to DC support, with Spain adopting a state-centric regulation with the government imposing responsibilities on HEIs to provide flexible academic paths, and the other countries presenting a laissez-faire approach with no formal structures (11). However, these approaches did not necessarily translate into clear differences in the presence, quality, and planned implementation of the studied DC areas. On the contrary, the cross-national variability observed in our results suggests that cultural and socioeconomic factors play a critical role. Kuettel et al. ( (40) demonstrated that dual career support systems are heavily influenced by welfare models and the historical trajectories of individual countries. Their study highlights how broader systemic factors, such as national investment in social welfare and the integration of sport within societal priorities, shape the availability and quality of dual career resources. For example, Serbian HEI experts reported the highest presence of services and implementation plans (Supplementary Table 1), which could reflect Serbia's status as an emerging country. Conversely, the lowest presence of services and plans in Italy and Spain might stem from a more precise understanding of what has been effectively implemented and a clearer awareness of the national legislative framework, compared to countries where the dual career topic is still emerging. Additionally, these findings may reflect higher expectations in countries accustomed to well-established services, leading to more critical evaluations. In Spain and Italy, S-As consistently provided lower evaluations than HEI experts, suggesting potentially higher expectations or a more critical perspective on the services received. Conversely, in Serbia, S-As often rated services higher than HEI experts, a trend occasionally mirrored in Slovenia, Romania, and Non-Find ME countries. These patterns highlight how cultural and socioeconomic contexts shape not only the availability of dual career services but also their perception. Overall, the findings of this study align with Kuettel et al. (40), confirming that the diversity in dual career provision reflects not only institutional practices but also the underlying welfare ideologies and cultural frameworks shaping each country's approach. The differences between HEI experts and S-As might be due to a limited familiarity and awareness of DC policies, programmes, initiatives, and documents available in the country and call for better communication and understanding of the legal DC framework that impacts on the available support structures (20, 41). Actually, improvements in DC do not necessarily derive from national policies but can also emerge from the cooperation of different stakeholders. In fact, the recent establishment of the Italian network of HEIs Unisport Italia fostered the cooperation with the Olympic (CONI), Paralympic (CIP) and the University Sport Federation (CUSI) to publish the Italian DC guidelines, the national online DC platform for S-As interested in enrolling at universities offering a suitable educational degree and a DC support (https://www.unisport-italia.it/dual-career/), and a relevant involvement of HEIs in ERASMUS + DC projects (41, 42). It is also possible to speculate that such a consolidate dialogue on DC determined the highest percentage of Italian respondents to the surveys. Finally, the European Commission is the driving force softly directing the development of the quality assurance of DC infrastructure, and a powerful actor in the evaluation processes through its funding, networks, indicators, data processes, and discourses (42).

The results of the present study highlight the importance of considering both institutional perspectives and the experiences of S-As in shaping effective DC policies. Specifically, the observed cross-national variability highlights the need for tailored policy approaches that reflect the unique cultural and socioeconomic contexts of each country. Policymakers should use these findings to align national strategies with European frameworks, such as the EU Guidelines on Dual Careers of Athletes, while adapting them to local needs to ensure fair access to DC provisions. Improving communication and resource visibility is essential, as gaps in knowledge and usage of DC services indicate the need for centralized platforms that provide clear information on available resources, guidelines, and best practices. These platforms could also offer tools for personalized academic planning, helping S-As better balance their commitments. Strengthening collaborations between HEIs, governments, sports organizations, and private entities is also critical. Such partnerships can drive the creation of innovative and sustainable DC ecosystems, support the co-creation of tailored solutions, promote resource sharing, and enhance the overall quality of DC support systems. Policymakers should also prioritize long-term investments in digital learning, flexible curricula, and financial support mechanisms to address current gaps and anticipate the evolving needs of S-As in a rapidly changing educational landscape. By addressing these key areas, policymakers and practitioners can develop strategies that are both practically viable and theoretically robust, fostering an environment where S-As can pursue dual careers successfully. These efforts will contribute to strengthening the European education area and ensuring that DC policies translate into tangible benefits for both institutions and S-As.

The present study has several strengths, including a comprehensive evaluation from both S-As and HEI experts’ perspectives, offering a well-rounded understanding of the current DC landscape. It also provides a cross-country comparison, highlighting both common trends and country-specific differences, which provides valuable insights for policymakers at various levels. Additionally, the study offers actionable insights by identifying specific areas for improvement in DC support and offering concrete suggestions for enhancing the quality and availability of services. However, the study also has limitations, firstly due to the sample size of different groups and the uneven representation of countries, with some nations being underrepresented, especially those with less universities. This imbalance might have influenced the findings, potentially amplifying some trends or underreporting unique challenges faced by underrepresented countries. Additionally, in countries with smaller sample sizes, extreme scores, either particularly high or low, might reflect the influence of outliers rather than a real trend across a given country. This could skew the overall interpretation of findings for these nations, highlighting the need for caution when generalizing results. As a result, the generalizability of the study's conclusions may be somewhat limited, particularly for nations with different socio-economic or cultural contexts. Future research should aim to address this limitation by ensuring more balanced representation across countries through targeted sampling strategies or collaborative international efforts. Secondly, the reliance on self-reported data could introduce bias, as participants' perceptions may not fully align with objective measures of support quality or availability. In some cases, the responses suggest a potential lack of knowledge regarding certain aspects of dual career support. As an example, HEI experts might have provided evaluations for National Legislation even in countries where such legislation does not formally exist, potentially confusing it with other documents, such as framework agreements or institutional guidelines. Furthermore, S-As often report the presence of services that experts do not recognize as available, likely because these services may be offered informally. This divergence underscores the complexity of dual career support systems, where formal policies and informal practices coexist, leading to potential inconsistencies in the perception and reporting of available resources. Thirdly, the study lacks longitudinal data, which limits its ability to assess the long-term impact and sustainability of dual career policies and support systems. For instance, some of the observed trends may reflect temporary conditions, such as recent policy changes, rather than stable patterns. This limitation also hinders our understanding of how dual career policies adapt to the evolving educational and athletic systems, as well as the broader socio-economic and political context. Addressing this gap in future longitudinal studies could involve tracking the implementation, sustainability, and effectiveness of specific initiatives, evaluating how policies influence the development of S-As over time, and assessing whether short-term benefits lead to long-term outcomes. Finally, the present study focuses exclusively on the perspectives of HEI experts and S-As. However, the absence of data from other relevant actors, such as parents, coaches, and policymakers, represents a limitation. These stakeholders play critical roles in shaping the dual career ecosystem, and their perspectives could provide valuable additional information. Future research should aim to include these voices to offer a more comprehensive understanding of the challenges and opportunities within dual career support systems.

## 5 Conclusions

To align to the DC priority of European sports policies and guidelines for pursuing the holistic development of elite athletes, a coordinated approach to DC support is required (6, 12, 14, 16, 18, 43). In conclusion, the findings from the present study underscore the complex and multifaceted nature of DC support for S-As across Europe. While logistic support is generally the most provided service, significant variations exist in quality and availability among different countries. The confirmation of logistic support as a prevalent service underscores the importance of maintaining and enhancing these resources. Future policies should focus on addressing the identified gaps, particularly in countries where support is less robust, to ensure equitable access for all S-As. In this regard, leveraging broader policy frameworks, such as the Bologna Process and ERASMUS + initiatives, could play an important role. The Bologna Process provides tools like micro-credentials and mutual recognition of qualifications, which can help harmonize educational pathways for S-As. Similarly, the ERASMUS + initiative offers funding opportunities for trans-national collaborations and innovative program development, which could be especially beneficial for resource-constrained HEIs. By aligning institutional strategies with these frameworks, HEIs and policymakers can create more cohesive and accessible dual career support systems across Europe. Notwithstanding, the discrepancies between the evaluations of S-As and HEI experts highlight the necessity of considering both perspectives to improve support systems effectively.

## Data Availability

The raw data supporting the conclusions of this article will be made available by the authors, without undue reservation.
