# Supplementary material for: A study on the implementation of dual career at European higher education institutions: the student-athletes' and experts' views
Source: Front Sports Act Living. 2025 Feb 25;7:1507951. doi: 10.3389/fspor.2025.1507951 (PMC11893579; doi:10.3389/fspor.2025.1507951)
Supplement: Supplementary file 1 [file Table1.docx]

**Supplementary table 1.** Frequency of occurrence (%) of dual career items, perceived quality, planned implementation, and year of implementation reported by University Experts.

|  |  | **Italy (n. 22)** | | **Romania (n. 7)** | | **Serbia (n. 4)** | | **Slovenia (n. 2)** | | **Spain (n. 6)** | | **Non-FINFD ME Countries (n. 4)** | |
| --- | --- | --- | --- | --- | --- | --- | --- | --- | --- | --- | --- | --- | --- |
|  |  | Presence | Implementation | Presence | Implementation | Presence | Implementation | Presence | Implementation | Presence | Implementation | Presence | Implementation |
| **Thematic areas** | **items** | (Quality) | (years) | Quality | (years) | (Quality) | (years) | (Quality) | (years) | (Quality) | (years) | (Quality) | (years) |
| Logistic support | Educational facilities | 95% (4.4 ± 0.7 pt) | 65% (2025 ± 1.3) | 100% (4.3 ± 1.1) | 71% (2025 ± 1.5) | 100% (3.3 ± 1.0) | 100% (2026 ± 1.3) | 100% (5.0) | 50% (2027 ± N/A) | 83% (3.8 ± 1.3) | 17% (2025 ± N/A) | 100% (4.5 ± 1.0) | 50% (2025 ± 1.4) |
|  | Sports facilities | 95% (3.9 ± 1.0 pt) | 83% (2026 ± 1.4) | 100% (3.7 ± 1.1) | 71% (2026 ± 1.6) | 100% (4.0 ± 1.4) | 100% (2027 ± 1.2) | 100% (5.0) | 0% (N/A) | 83% (3.0 ± 1.2) | 17% (2025 ± N/A) | 75% (4.7 ± 0.6) | 75% (2026 ± 2.3) |
|  | Economic investments in facilities | 91% (3.7 ± 0.9 pt) | 74% (2026 ± 1.1) | 100% (4.0 ± 0.8) | 71% (2027 ± 1.6) | 100% (2.5 ± 0.6) | 100% (2026 ± 1.3) | 100% (2.5 ± 0.7) | 0% (N/A) | 83% (4.4 ± 1.3) | 33% (2025 ± 0.0) | 100% (3.8 ± 1.3) | 75% (2026 ± 2.3) |
|  | Accommodation for S-As | 37% ( (3.7 ± 0.9 pt) | 52% (2026 ± 1.2) | 100% (4.0 ± 1.2) | 71% (2026 ± 1.1) | 50% (4.0 ± 1.4) | 75% (2027 ± 1.5) | 100% (3.0 ± 1.4) | 0% (N/A) | 50% (2.0) | 0% (N/A) | 100% (4.3 ± 1.0) | 75% (2026 ± 0.6) |
| Assistance/tutorship | Tutorship/mentorship | 96% (4.6 ± 0.7 pt) | 57% (2025 ± 1.0) | 71% (3.5 ± 1.2) | 71% (2026 ± 2.0) | 75% (3.0 ± 1.7) | 100% (2027 ± 1.2) | 50% (5.0) | 0% (N/A) | 100% (4.2 ± 1.2) | 17% (2025 ± 0.0) | 100% (4.3 ± 0.8) | 50% (2026 ± 0.7) |
|  | Individual programmes | 96% (4.4 ± 0.8 pt) | 52% (2026 ± 1.3) | 71% (2.8 ± 1.1) | 43% (2026 ± 2.3) | 50% (2.0) | 100% (2027 ± 1.2) | 100% (3.5 ± 1.5) | 0% (N/A) | 100% (4.4 ± 0.9) | 17% (2025 ± 0.0) | 100% (3.8 ± 0.8) | 50% (2026 ± 0.7) |
|  | Integration of academic department, sports or professional services | 68% (3.7 ± 1.0 pt) | 52% (2026 ± 1.2) | 57% (3.3 ± 1.3) | 43% (2026 ± 2.3) | 75% (2.3 ± 0.6) | 100% (2027 ± 1.2) | 100% (3.5 ± 1.5) | 0% (N/A) | 83% (3.6 ± 1.4) | 17% (2027 ± 2.0) | 100% (3.3 ± 0.8) | 100% (2026 ± 1.1) |
|  | Psychologic support | 77% (3.9 ± 1.3 pt) | 48% (2026 ± 1.1) | 71% (3.4 ± 1.3) | 57% (2027 ± 1.3) | 75% (3.5 ± 2.1) | 100% (2027 ± 1.2) | 100% (3.5 ± 1.5) | 0% (N/A) | 67% (2.7 ± 1.2) | 50% (2027 ± 1.3) | 100% (2.5 ± 0.5) | 100% (2026 ± 1.2) |
|  | Dual career proactive programmes | 77% (3.4 ± 1.3 pt) | 52% (2026 ± 1.1) | 57% (3.3 ± 1.3) | 57% (2027 ± 1.3) | 50% (3.5 ± 1.5) | 100% (2027 ± 1.2) | 50% (3.0) | 0% (N/A) | 50% (3.5 ± 1.0) | 17% (2025 ± 0.0) | 100% (3.3 ± 0.5) | 75% (2027 ± 1.5) |
| Curricula requirements | Individual study plan | 73% (3.9 ± 1.0) | 57% (2025 ± 1.0) | 86% (3.7 ± 0.8) | 57% (2026 ± 1.8) | 0% (-) | 75% (2027 ± 1.0) | 50% (5.0) | 0% (N/A) | 67% (3.8 ± 1.5) | 17% (2025 ± N/A) | 75% (4.0 ± 1.7) | 50% (2025 ± 0.7) |
|  | Distance learning | 59% (3.8 ± 1.2 pt) | 39% (2025 ± 1.2) | 86% (2.7 ± 0.8) | 57% (2026 ± 2.1) | 50% (4.0 ± 1.4) | 75% (2027 ± 1.0) | 100% (3.5 ± 1.5) | 0% (N/A) | 67% (3.0 ± 1.4) | 17% (2025 ± N/A) | 100% (4.3 ± 1.5) | 75% (2026 ± 1.2) |
|  | Recognition of ECTS for the sport career | 59% (3.8 ± 1.2 pt) | 39% (2025 ± 1.4) | 29% (2.7 ± 0.8) | 71% (2026 ± 1.8) | 75% (3.3 ± 0.6) | 75% (2027 ± 1.0) | 0% (-) | 0% (N/A) | 67% (5.0) | 0% (N/A) | 75% (3.7 ± 1.5) | 75% (2027 ± 1.0) |
|  | Untraditional learning strategies | 32% (3.3 ± 1.0 pt) | 33% (2026 ± 2.0) | 14% (4.0) | 71% (2026 ± 1.8) | 100% (3.0 ± 0.0) | 100% (2027 ± 1.0) | 50% (2.0) | 0% (N/A) | 33% (2.0) | 17% (2024 ± N/A) | 75% (3.3 ± 1.5) | 75% (2026 ± 1.5) |
| Social support | Publicity for student-athletes representing the university | 86% (3.9 ± 1.1 pt) | 74% (2025 ± 1.4) | 88% (3.5 ± 1.4) | 71% (2025 ± 1.9) | 75% (4.7 ± 0.6) | 100% (2026 ± 1.6) | 100% (3.0) | 0% (N/A) | 83% (3.2 ± 1.1) | 50% (2025 ± 0.6) | 100% (4.0 ± 0.8) | 75% (2025 ± 1.2) |
|  | Local/international seminars, workshop, meeting on dual career issues | 67% (3.5 ± 1.2 pt) | 61% (2025 ± 1.4) | 71% (4.0 ± 1.0) | 57% (2026 ± 2.1) | 100% (2.7 ± 0.6) | 100% (2026 ± 1.6) | 50% (4.0) | 0% (N/A) | 50% (3.7 ± 1.2) | 33% (2025 ± 0.7) | 100% (4.0 ± 0.8) | 100% (2025 ± 1.0) |
|  | Institutional dual career committee | 63% (4.1 ± 1.1 pt) | 61% (2025 ± 1.5) | 57% (3.0) | 57% (2026 ± 2.1) | 100% (2.7 ± 0.6) | 75% (2026 ± 2.0) | 50% (4.0) | 0% (N/A) | 50% (3.0 ± 1.7) | 33% (2025 ± 0.7) | 50% (4.3 ± 0.6) | 100% (2024 ± 0.5) |
|  | Publicity of student-athletes' characteristics for labor market | 59% (3.6 ± 0.9 pt.) | 57% (2025 ± 1.3) | 43% (2.3 ± 0.6) | 57% (2026 ± 2.1) | 75% (3.7 ± 1.2) | 100% (2026 ± 1.6) | 50% (3.0) | 0% (N/A) | 50% (3.0 ± 1.7) | 33% (2024 ± 0.0) | 100% (3.5 ± 0.6) | 75% (2026 ± 1.5) |
|  | Peer to peer support | 73% (3.7 ± 1.0 pt) | 43% (2024 ± 1.5) | 100% (3.7 ± 1.1) | 71% (2026 ± 2.1) | 100% (2.7 ± 0.6) | 75% (2026 ± 2.0) | 50% (5.0) | 0% (N/A) | 50% (3.5 ± 1.5) | 33% (2025 ± 0.0) | 100% (3.3 ± 1.0) | 100% (2026 ± 1.9) |
|  | Seminars, workshop, meetings with parents and coaches | 40% (2.9 ± 0.8 pt) | 43% (2024 ± 1.4) | 57% (3.0 ± 0.7) | 71% (2026 ± 2.1) | 75% (3.7 ± 1.2) | 100% (2026 ± 1.6) | 50% (2.0) | 0% (N/A) | 50% (2.5 ± 0.7) | 17% (2025 ± N/A) | 100% (4.0 ± 0.8) | 100% (2026 ± 1.3) |
| Financial support | Scholarship for S-As | 46% (4.5 ± 1.0 pt) | 39% (2025 ± 1.3) | 43% (5.0) | 43% (2025 ± 2.1) | 0% (-) | 100% (2026 ± 1.9) | 0% (-) | 0% (N/A) | 50% (4.0 ± 1.7) | 17% (2024 ± N/A) | 100% (4.0 ± 1.2) | 100% (2024 ± 0.0) |
|  | Remission of tuition fees for S-As | 64% (3.9 ± 1.0 pt | 39% (2025 ± 1.6) | 57% (4.0 ± 0.8) | 43% (2025 ± 2.1) | 50% (2.5 ± 1.9) | 100% (2026 ± 1.9) | 0% (-) | 0% (N/A) | 33% (3.0 ± 1.4) | 17% (2024 ± N/A) | 75% (3.7 ± 1.2) | 75% (2025 ± 0.6) |
|  | Other forms of financial support | 36% (3.8 ± 1.2 pt) | 26% (2026 ± 1.6) | 29% (4.0 ± 1.4) | 43% (2025 ± 2.1) | 75% (1.8 ± 1.3) | 75% (2026 ± 2.0) | 50% (5.0) | 0% (N/A) | 33% (3.3 ± 1.2) | 33% (2025 ± 0.7) | 75% (3.7 ± 1.2) | 100% (2024 ± 0.5) |
|  | Salary | 9% (3.0 ± 1.4 pt) | 13% (2026 ± 1.7) | 0% (-) | 43% (2025 ± 2.1) | 100% (3.0) | 50% (2026 ± 2.8) | 0% (-) | 0% (N/A) | 0% (-) | 0% (N/A) | 25% (2.0) | 0% (N/A) |
| Other supports/DC policies | Sport observatory of the application of the dual career statute | 72% (4.0 ± 1.1 pt) | 52% (2024 ± 1.3) | 71% (3.8 ± 1.1) | 43% (2025 ± 2.3) | 100% (2.7 ± 0.5) | 100% (2027 ± 1.0) | 0% (-) | 0% (N/A) | 80% (3.0) | 33% (2025 ± 0.7) | 100% (4.0 ± 0.8) | 75% (2025 ± 1.0) |
|  | National legislation | 68% (3.3 ± 1.0 pt) | 35% (2026 ± 1.8) | 100% (3.0 ± 1.0) | 43% (2025 ± 2.3) | 100% (3.0 ± 0.8) | 100% (2027 ± 1.0) | 100% (2.5 ± 0.7) | 0% (N/A) | 80% (3.6 ± 1.4) | 17% (2027 ± N/A) | 100% (3.2 ± 1.3) | 100% (2027 ± 1.4) |
|  | Special access contingent for actual or former elite athletes | 45% (3.3 ± 1.0 pt) | 35% (2025 ± 1.7) | 57% (3.0 ± 1.0) | 43% (2025 ± 2.3) | 100% (2.7 ± 1.0) | 100% (2027 ± 1.0) | 50% (5.0) | 0% (N/A) | 80% (4.0 ± 1.4) | 0% (N/A) | 100% (3.0 ± 1.4) | 100% (2025 ± 1.0) |
