# Supplementary material for: A study on the implementation of dual career at European higher education institutions: the student-athletes' and experts' views
Source: Front Sports Act Living. 2025 Feb 25;7:1507951. doi: 10.3389/fspor.2025.1507951 (PMC11893579; doi:10.3389/fspor.2025.1507951)
Supplement: Supplementary file 2 [file Table2.docx]

**Supplementary table 2.** Frequency of occurrence (%) of dual career items and perceived quality reported by Student-Athletes.

|  |  | **Italy (n. 122)** | | **Romania (n. 52)** | | **Serbia (n. 44)** | | **Slovenia (n.17)** | | **Spain (n. 58)** | | **Non-FINFD ME Countries (n. 28)** | |  |  |  |
| --- | --- | --- | --- | --- | --- | --- | --- | --- | --- | --- | --- | --- | --- | --- | --- | --- |
|  |  | Presence | Quality | Presence | Quality | Presence | Quality | Presence | Quality | Presence | Quality | Presence | Quality |  |  |  |
| **Thematic areas** | **items** | (%) | (pt) | (%) | (pt) | (%) | (pt) | (%) | (pt) | (%) | (pt) | (%) | (pt) | ***F*** | ***p*** | ***η²*** |
| Logistic support | educational facilities | 93 | 3.4 ± 1.1 | 98 | 4.0 ± 1.1^a^ | 100 | 3.4 ± 1.1 | 94 | 3.2 ± 1.1 | 90 | 3.6 ± 1.3 | 100 | 3.9 ± 1.1 | 3.23 | 0.01 | 0.05 |
|  |  |  |  |  |  |  |  |  |  |  |  |  |  |  |  |  |
|  | sports facilities | 82 | 2.9 ± 1.3 | 94 | 3.7 ± 1.1^a,b^ | 98 | 3.4 ± 1.1 | 94 | 3.2 ± 1.1 | 79 | 2.9 ± 1.3 | 82 | 3.1 ± 1.4 | 4.14 | 0.002 | 0.06 |
|  | economic investments in facilities | 90 | 2.9 ± 1.1 | 87 | 3.5 ± 0.8 | 91 | 3.1 ± 1.2 | 88 | 2.7 ± 1.1 | 83 | 2.8 ± 1.3 | 86 | 2.9 ± 1.2 | 2.61 | 0.03 | 0.05 |
|  | accommodation for S-As | 71 | 2.7 ± 1.4 | 88 | 3.6 ± 1.3^a,b,c,d^ | 84 | 3.0 ± 1.3^b^ | 65 | 2.3 ± 1.2 | 50 | 1.9 ± 1.2 | 82 | 2.9 ± 1.3 | 10.01 | <0.001 | 0.13 |
| Assistance/tutorship | Tutorship/mentorship | 83 | 3.2 ± 1.4 | 77 | 3.3 ± 1.6 | 95 | 3.4 ± 1.3 | 88 | 2.8 ± 1.2 | 91 | 3.5 ± 1.3 | 89 | 3.3 ± 1.2 | 0.98 | 0.44 | 0.01 |
|  | Individual programmes | 80 | 3.0 ± 1.4 | 85 | 3.3 ± 1.4 | 84 | 3.2 ± 1.4 | 82 | 2.6 ± 1.1 | 67 | 2.5 ± 1.4 | 85 | 3.3 ± 1.4 | 1.22 | 0.32 | 0.03 |
|  | Integration of academic department, sports or professional services | 78 | 2.8 ± 1.4 | 85 | 3.2 ± 1.4 | 86 | 3.2 ± 1.4 | 88 | 2.5 ± 1.0 | 60 | 2.3 ± 1.4 | 93 | 3.1 ± 1.1 | 1.32 | 0.26 | 0.02 |
|  | Psychologic support | 64 | 2.5 ± 1.4 | 73 | 2.9 ± 1.5^b^ | 84 | 3.3 ± 1.5^b^ | 88 | 2.6 ± 1.1 | 53 | 2.0 ± 1.2 | 79 | 2.7 ± 1.3 | 4.90 | <0.001 | 0.07 |
|  | Dual career proactive programmes | 76 | 2.8 ± 1.3 | 77 | 3.0 ± 1.5 | 84 | 3.2 ± 1.5 | 94 | 2.6 ± 0.9 | 57 | 2.1 ± 1.2 | 82 | 2.9 ±1.2 | 1.44 | 0.22 | 0.04 |
| Curricula requirements | Individual study plan | 72 | 3.1 ± 1.5 | 81 | 2.3 ± 1.6 | 82 | 1.0 ± 0.0 | 76 | 3.0 ± 2.8 | 69 | 2.8 ± 1.8 | 79 | 3.3 ± 2.1 | 1.62 | 0.16 | 0.03 |
|  | Distance learning | 73 | 2.6 ± 1.4 | 75 | 2.4 ± 1.5 | 86 | 2.5 ± 1.9 | 82 | 3.5 ± 2.1 | 74 | 2.3 ± 1.5 | 86 | 4.3 ± 1.5 | 1.11 | 0.36 | 0.02 |
|  | Recognition of ECTS for the sport career | 69 | 2.6 ± 1.6 | 83 | 2.3 ± 1.6 | 82 | 2.8 ± 1.3 | 88 | 1.0 ± 0.0 | 71 | 3.7 ± 2.1 | 82 | 3.0 ± 1.8 | 1.32 | 0.26 | 0.02 |
|  | Untraditional learning strategies | 66 | 1.7 ± 1.2 | 77 | 2.4 ± 1.5 | 84 | 3.0 ± 0.0 | 88 | 1.5 ± 0.7 | 74 | 1.3 ± 0.5 | 75 | 3.3 ± 1.5 | 2.73 | 0.02 | 0.04 |
| Social support | Publicity for student-athletes representing the university | 67 | 2.5 ± 1.3 | 69 | 2.8 ± 1.5 | 91 | 3.3 ± 1.3 | 94 | 3.1 ± 1.0 | 66 | 2.5 ± 1.5 | 82 | 3.1 ± 1.3 | 3.60 | <0.001 | 0.05 |
|  | Local/international seminars, workshop, meeting on dual career issues | 73 | 2.5 ± 1.2 | 67 | 2.6 ± 1.5 | 91 | 3.2 ± 1.3 | 94 | 2.8 ± 1.0 | 57 | 2.2 ± 1.3 | 82 | 2.9 ± 1.1 | 3.42 | 0.007 | 0.05 |
|  | Institutional dual career committee | 72 | 2.6 ± 1.3^b^ | 58 | 2.4 ± 1.6 | 89 | 3.3 ± 1.4^a,e^ | 82 | 2.6 ± 1.0 | 50 | 2.0 ± 1.2 | 68 | 2.5 ± 1.3 | 5.51 | <0.001 | 0.07 |
|  | Publicity of student-athletes' characteristics for labor market | 62 | 2.3 ± 1.3 | 63 | 2.5 ± 1.6 | 89 | 3.1 ± 1.3 | 88 | 2.7 ± 0.9 | 53 | 2.2 ± 1.4 | 75 | 2.7 ± 1.2 | 3.20 | 0.01 | 0.05 |
|  | Peer to peer support | 67 | 2.4 ± 1.2 | 63 | 2.6 ± 1.6 | 86 | 3.2 ± 1.4^a,b,d^ | 94 | 3.1 ± 1.0 | 53 | 2.2 ± 1.3 | 64 | 2.5 ± 1.3 | 3.72 | 0.004 | 0.05 |
|  | Seminars, workshop, meetings with parents and coaches | 57 | 2.1 ± 1.2 | 60 | 2.6 ± 1.6 | 86 | 3.3 ± 1.4^a,b^ | 82 | 2.7 ± 1.1 | 48 | 2.0 ± 1.2 | 61 | 2.1 ± 1.0 | 6.90 | <0.001 | 0.11 |
| Financial support | Scholarship for student-athletes | 63 | 2.4 ± 1.3 | 81 | 3.1 ± 1.5^a^ | 89 | 2.8 ± 1.7^a^ | 59 | 2.3 ± 1.4 | 69 | 2.4 ± 1.6 | 61 | 2.7 ± 1.6 | 4.05 | 0.002 | 0.06 |
|  | Remission of tuition fees for student-athletes | 66 | 2.3 ± 1.5 | 56 | 2.3 ± 1.5 | 91 | 2.8 ± 1.7^b,e^ | 59 | 2.2 ± 1.2 | 57 | 2.1 ± 1.1 | 71 | 3.0 ± 1.5 | 4.08 | 0.002 | 0.05 |
|  | Other forms of financial support | 57 | 2.0 ± 1.5 | 60 | 2.6 ± 1.6 | 86 | 2.5 ± 1.9^b,c^ | 59 | 2.1 ± 1.1 | 60 | 2.2 ± 1.5 | 61 | 2.6 ± 1.5 | 1.11 | 0.36 | 0.02 |
|  | Salary | 38 | 2.4 ± 1.3 | 46 | 3.1 ± 1.5^b^ | 82 | 2.8 ± 1.7^a,b,c,d,e^ | 35 | 2.3 ± 1.4 | 22 | 2.4 ± 1.6 | 39 | 2.7 ± 1.6 | 10.28 | <0.001 | 0.17 |
| Other supports/DC policies | Sport observatory of the application of the dual career statute | 65 | 2.4 ± 1.3 | 56 | 2.3 ± 1.4 | 89 | 3.1 ± 1.3^a,b,e^ | 88 | 2.6 ± 0.9 | 57 | 2.1 ± 1.2 | 68 | 2.7 ± 1.4 | 3.93 | 0.003 | 0.06 |
|  | National legislation | 64 | 2.3 ± 1.2 | 56 | 2.2 ± 1.3 | 93 | 3.2 ± 1.1 ^a,b,d,e^ | 94 | 2.6 ± 0.9 | 67 | 2.4 ± 1.2 | 68 | 2.4 ± 1.2 | 5.01 | <0.001 | 0.07 |
|  | Special access contingent for actual or former elite athletes | 57 | 2.2 ± 1.2 | 52 | 2.2 ± 1.4 | 89 | 3.2 ± 1.2 ^a,b,d,e^ | 94 | 2.6 ± 0.8 | 67 | 2.5 ± 1.3 | 68 | 2.4 ± 1.2 | 4.77 | <0.001 | 0.07 |

**Table 3.** Significant differences at Tukey's test (*p* < 0.05) are reported. ^a^indicates scores significantly higher than those of Italian student-athletes (S-As) scores. ^b^indicates scores significantly higher than those of Spanish S-As. ^c^indicates scores significantly higher than those of Slovenian S-As scores. ^d^indicates scores significantly higher than those of Non-Find ME S-As. ^e^indicates scores significantly higher than those of Romanian S-As.
